# Kukoamine B promotes TLR4-independent lipopolysaccharide uptake in murine hepatocytes

**DOI:** 10.18632/oncotarget.11292

**Published:** 2016-08-15

**Authors:** Dong Yang, Xinchuan Zheng, Ning Wang, Shijun Fan, Yongjun Yang, Yongling Lu, Qian Chen, Xin Liu, Jiang Zheng

**Affiliations:** ^1^ Medical Research Center, Southwest Hospital, Third Military Medical University, Chongqing, China

**Keywords:** lipopolysaccharide, kukoamine B, hepatocytes, asialoglycoprotein receptor, Immunology and Microbiology Section, Immune response, Immunity

## Abstract

Free bacterial lipopolysaccharide (LPS) is generally removed from the bloodstream through hepatic uptake via TLR4, the LPS pattern recognition receptor, but mechanisms for internalization and clearance of conjugated LPS are less clear. Kukoamine B (KB) is a novel cationic alkaloid that interferes with LPS binding to TLR4. In this study, KB accelerated blood clearance of LPS. KB also enhanced LPS distribution in the hepatic tissues of C57 BL/6 mice, along with LPS uptake in primary hepatocytes and HepG2 cells. By contrast, KB inhibited LPS internalization in Kupffer and RAW 264.7 cells. Loss of TLR4 did not affect LPS uptake into KB-treated hepatocytes. We also detected selective upregulation of the asialoglycoprotein receptor (ASGPR) upon KB treatment, and ASGPR colocalized with KB in cultured hepatocytes. Molecular docking showed that KB bound to ASGPR in a manner similar to GalNAc, a known ASGPR agonist. GalNAc dose-dependently reduced KB internalization, suggesting it competes with KB for ASGPR binding, and ASGPR knockdown also impaired LPS uptake into hepatocytes. Finally, while KB enhanced LPS uptake, it was protective against LPS-induced inflammation and hepatocyte injury. Our study provides a new mechanism for conjugated LPS hepatic uptake induced by the LPS neutralizer KB and mediated by membrane ASGPR binding.

## INTRODUCTION

Recognition of lipopolysaccharide (LPS), a glycolipid compound that constitutes much of the outer membrane in Gram-negative bacteria, is essential for triggering the host immune response [1–3]. LPS is generally recognized *via* its pattern recognition receptor, TLR4, and induces pro-inflammatory MyD88-dependent or -independent signaling pathways [4–6]. Whereas essential TLR4 activation is required to facilitate infection control, excessive TLR4 stimulation by LPS may result in serious consequences, such as sepsis, multiple organ dysfunction (MODS) and shock [7, 8]. The physical state of bloodstream LPS, either in free form or in complex with LPS binding compounds, determines its ability to induce systemic inflammation [9–12]. Lipoprotein-bound LPS exhibits much weaker activity than free LPS in stimulating macrophages to release pro-inflammatory cytokines like TNF-α and IL-6. This is presumably due to blockage of the LPS lipid A moiety-TLR4 interaction by lipoproteins [13, 14]. Similar results have been observed in LPS conjugated to BPI, LBP or LL-37 [15, 16]. Additionally, a group of exogenous agents derived from natural products or antimicrobial peptides can neutralize LPS, and may have potential as anti-sepsis therapies [17, 18]. However, there are few reports describing the possible roles of such drugs in accelerating LPS uptake and removal.

The liver is an important organ in bacterial LPS absorption and metabolism, and LPS is reportedly quickly enriched in murine liver tissues after intravenous injection [19, 20]. There are generally four types of hepatic cells, including parenchymal hepatocytes (HCs), non-parenchymal Kupffer cells (KCs), liver sinusoidal endothelial cells (LSECs) and stellate cells (HSCs) [21, 22]. In particular, KCs were identified as the predominant cell type for hepatic LPS uptake [10, 23], although evidence suggests that LPS can also be efficiently internalized by HCs or LSECs [20, 24]. Circulating LPS is commonly conjugated by carrier proteins or other neutralizing agents, complicating the process of LPS adsorption and metabolism.

Free LPS uptake is receptor mediated and TLR4 is best known to mediate uptake in intrahepatic cells. Deng, *et al.* found that TLR4 was functionally necessary for endotoxin and bacteria removal by the liver during sepsis [19]. However, excessive TLR4-dependent internalization may cause cell damage as inflammatory signaling pathways are activated concomitantly, leading to excessive release of inflammatory factors such as TNF-α and IL-6. Other receptors, like ASGPR, CD14, CD11b/CD18, SR and LDL, are therefore also utilized by hepatic cells to mediate LPS uptake and avoid excessive TLR4 activation. For example, HDL, apolipoprotein A-I and α1-acid glycoprotein may facilitate liver uptake and removal of LPS while inhibiting inflammation [25, 26].

LPS is the key factor in triggering sepsis, which may be prevented or attenuated if LPS is effectively neutralized or rapidly removed from the blood stream [27]. We identified kukoamine B (KB), a cationic alkaloid from the root of *Cortex Lycii*, as a novel potential LPS neutralizing agent. Our preliminary work showed that KB directly neutralized LPS activity. KB also effectively inhibits LPS-induced inflammation and improves survival in mouse sepsis models [28, 29]. However, it remains unclear whether KB affects LPS distribution and how it mediates LPS uptake and removal within the host. The present study explores the organs, cells and receptors participating in KB-mediated LPS uptake, and describes the possible biological mechanism of KB activity in treating sepsis.

## RESULTS

### KB decreases LPS levels in mouse peripheral blood and enhances LPS accumulation in hepatic tissues

**Figure 1:**
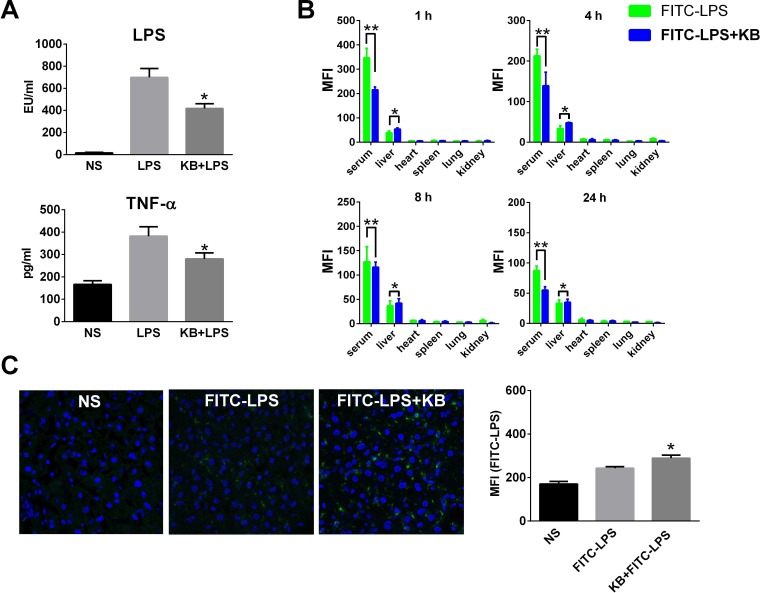
KB increases serum clearance of LPS and enhances hepatic LPS uptake in C57 BL/6 mice **A.** Mice were injected with normal saline (NS) or LPS (1 mg/kg) preincubated with NS or KB (3.6 mg/kg) at 37°C for 30 min. Serum levels of LPS and TNF-α were detected 4 h after injection (*n* = 3). **B.** Mice were injected with FITC-LPS (1 mg/kg) preincubated with NS or with KB (3.6 mg/kg). Serum and homogenates from liver, heart, spleen, lung and kidney were obtained at 1, 4, 8 and 24 h and FITC-LPS fluorescence was detected. *P < 0.05, **P < 0.01. **C.** C57 BL/6 mice were treated as in **B.** for 1 h, frozen liver tissue sections were prepared and FITC-LPS fluorescence was detected by confocal microscopy (objective×40). FITC-LPS fluorescence in whole liver tissues is expressed as means ± SD (*n* = 3). *P < 0.05 *vs.* FITC-LPS group.

KB was identified in our previous study as directly binding LPS *in vitro* [29]. In the present study, KB reduced free LPS in the serum of LPS-injected mice. It also inhibited elevation of serum TNF-α due to LPS injection (Figure 1A). Fluorescein isothiocyanate (FITC)-labeled LPS (FITC-LPS) was injected intravenously with or without preincubation with KB, and serum and tissue fluorescence was monitored. Results demonstrated that free FITC-LPS was gradually cleared in serum and then detected mainly in liver homogenates (Figure 1B). Serum LPS fluorescence decreased more quickly when FITC-LPS was preincubated with KB. In particular, KB co-injection selectively enhanced FITC-LPS accumulation in liver, but did not affect its distribution in other organs. In direct fluorescence imaging detection, we observed increased FITC-LPS distribution in liver sections of mice co-injected with KB (Figure 1C). These data together indicated that KB not only inhibited LPS bioactivity, but also increased its clearance by enhancing hepatic uptake.

### Hepatocytes selectively mediate increased LPS uptake induced by KB in a TLR4 independent manner

**Figure 2:**
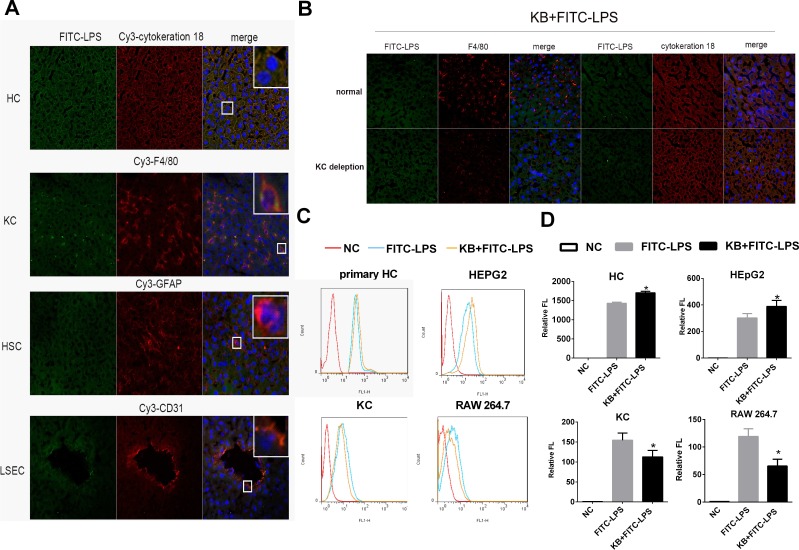
KB promotes LPS absorption by HCs and inhibits LPS uptake by KCs **A.** C57 BL/6 mice were injected with FITC-LPS (1 mg/kg) preincubated with KB (3.6 mg/kg) for 1h. Colocalization of FITC-LPS (green) with cell markers for HCs, KCs, HSCs and LSECs (red) was detected in frozen liver sections by confocal microscopy (objective×40). Nuclei were stained with DAPI (blue). **B.** C57 BL/6 mice were injected with NS or Clodronate liposomes for KC depletion, followed by 1 mg/kg FITC-LPS preincubated with 3.6 mg/kg KB. Hepatic uptake of LPS and its colocalization with HCs and KCs in liver sections were detected. **C.** & **D.** Primary HCs (2×10^5^/ml), HepG2 (2×10^5^/ml) cells, primary KCs (1×10^6^/ml) and RAW 264.7 cells (1×10^6^/ml) were treated with FITC-LPS (1 μg/ml) preincubated with H_2_O or KB (15 μM) for 1h. LPS uptake was detected by flow cytometry and expressed as relative fluorescence (Relative FL). *P < 0.05 *vs* FITC-LPS.

As KB enhanced LPS accumulation in liver, we analyzed the types of hepatic cells involved in LPS uptake. Free FITC-LPS predominantly colocalized with cytokeratin-18 (HCs) and F4/80 (KCs) 1 h after intravenous injection (Figure 2A). To determine whether HCs and KCs were both required for conjugated LPS uptake, mouse KCs were depleted by injection of clodronate liposomes. the injection caused complete loss of F4/80 antibody staining in hepatic liver sections, whereas cytokeratin-18 staining remained unaltered, indicating that hepatocytes were not affected (Figure S1). Despite Kupffer cell depletion, hepatic uptake of LPS preincubated with KB was still enhanced in the liver, suggesting KCs might be not the necessary cell type (Figure 2B). To further confirm that HCs were selectively required for KB-mediated LPS uptake, murine primary HCs and KCs were isolated and LPS uptake was measured *in vitro*. KB consistently enhanced FITC-LPS uptake in cultured primary HCs and suppressed LPS internalization in isolated KCs (Figure 2C-2D). KB also promoted LPS uptake in the hepatocyte-like cell line, HepG2, and decreased LPS absorption in the murine macrophage-like cell line, RAW 264.7 (Figure 2C-2D). Moreover, KB well colocalized with LPS in cultured primary hepatocytes (Figure S2).

**Figure 3:**
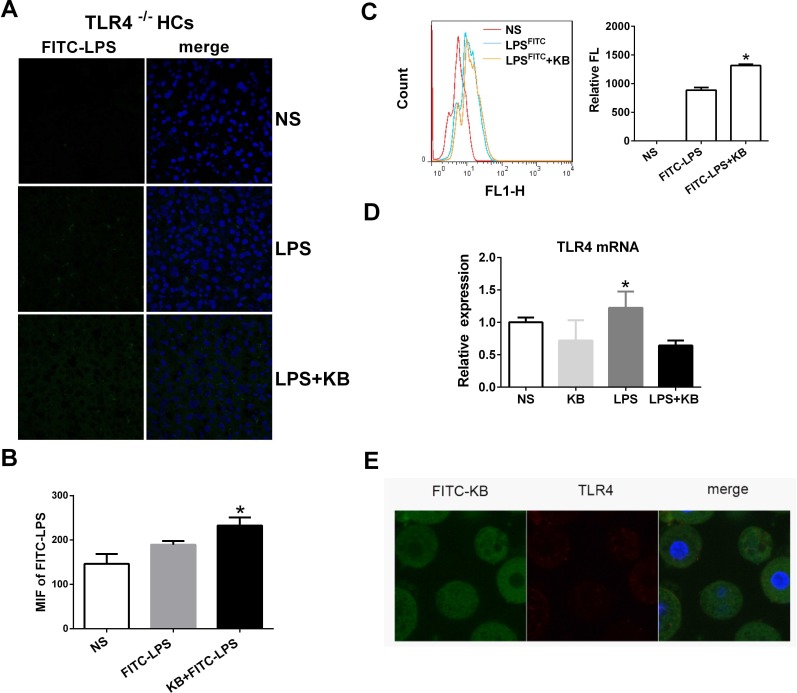
KB induced LPS uptake in hepatocytes independent of TLR4 **A.** TLR4^−/−^ mice were injected with NS and FITC-LPS (1 mg/kg) preincubated with NS or KB (3.6 mg/kg) for 1 h. Distribution of FITC-LPS on frozen liver sections was detected by confocal microscopy. **B.** FITC-LPS fluorescence in whole liver tissues is expressed as means ± SD (*n* = 3). *P < 0.05 *vs.* FITC-LPS. **C.** FITC-LPS uptake in HCs isolated from TLR4^−/−^ mice was analyzed by flow cytometry and expressed as relative fluorescence (Relative FL). *P < 0.05 *vs* FITC-LPS. **D.** HCs from WT C57BL/6 mice were treated with LPS (1 μg/ml) preincubated with or without KB (15 μM) for 4 h. TLR4 expression was detected by real-time PCR. **E.** HCs (2×10^5^/ml) from WT mice were treated with FITC-KB (15 μM) for 30 min. KB (green) and TLR4 (red) colocalization was detected by immunofluorescence.

TLR4 is the primary recognition receptor for LPS in hepatic cells, and we investigated whether or not LPS uptake following KB treatment was TLR4 dependent. Distributions of LPS preincubated with KB were similar in WT and TLR4^−/−^ mouse liver sections, suggesting that TLR4 deficiency did not affect KB-mediated LPS uptake (Figure 3A-3B). In addition, cellular uptake of free LPS was impaired in primary hepatocytes isolated from TLR4^−/−^ mice (data not shown). KB promoted LPS uptake in hepatocytes, even in the absence of TLR4 (Figure 3C), and did not alter TLR4 expression in WT hepatocytes, with or without LPS (Figure 3D). KB was also unable to colocalize with TLR4 in WT hepatocytes (Figure 3E). These data indicated that TLR4 might not be required to mediate LPS uptake in hepatocytes induced by KB.

### ASGPR is selectively upregulated by KB in hepatocytes and mediates LPS uptake

**Figure 4:**
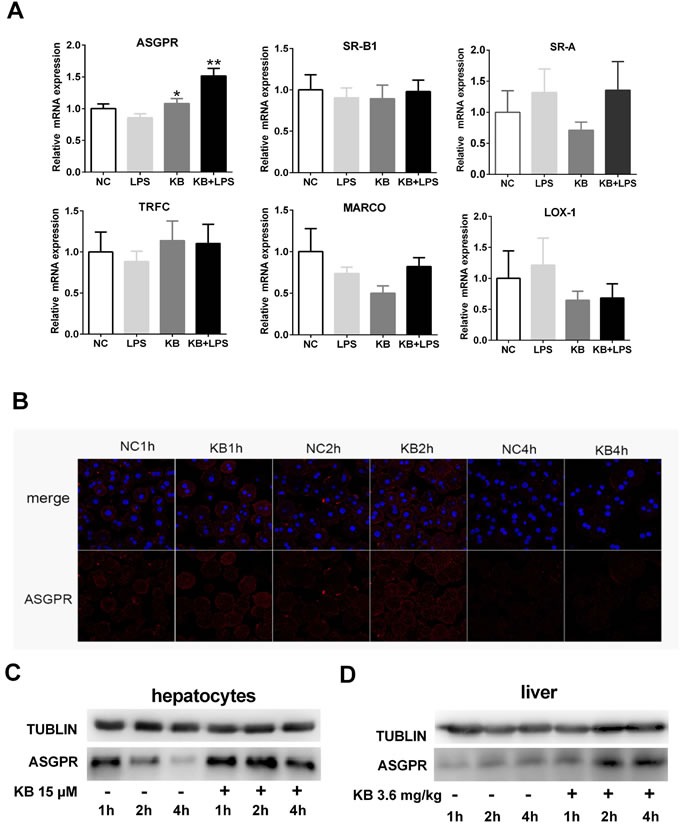
KB upregulates ASGPR in hepatocytes and mouse liver tissues **A.** Murine primary hepatocytes were untreated (NC) or treated with LPS (1 μg/ml), KB (15 μM) or LPS preincubated with KB for 4 h. Real-time PCR was used to detect ASGPR, MARCO, SR-A, SR-B1, TFRC and LOX-1 expression. *P < 0.05, **P < 0.01 *vs.* NC. **B.** & **C.** Primary hepatocytes isolated from WT C57BL/6 mice were untreated (NC) or treated with KB (15 μM) for 1, 2 or 4 h. ASGPR expression was detected by immunofluorescence **B.** or immunoblotting **C. D.** C57 BL/6 mice were injected with NS or KB (3.6 mg/kg) for 1, 2 or 4 h. ASGPR expression in liver homogenates as detected by immunoblotting, with tubulin as an internal control.

As TLR4 was not required for LPS uptake induced by KB, we screened other internalizing receptors that may be involved in LPS binding and internalization. KB selectively increased ASGPR mRNA expression in cultured primary HCs after treatment for 4 h. Although free LPS treatment did not upregulate ASGPR expression, KB still effectively increased ASGPR levels in the presence of LPS. In contrast, KB did not affect the expression of scavenger receptors (MARCO, SR-A and SR-B1), transferrin receptor (TFRC) or LOX-1, with or without LPS (Figure 4A). KB also upregulated ASGPR protein levels in primary HCs in a time-dependent manner (Figure 4B-4C), and in C57 BL/6 mice, KB injection upregulated ASGPR in liver tissues (Figure 4D).

**Figure 5:**
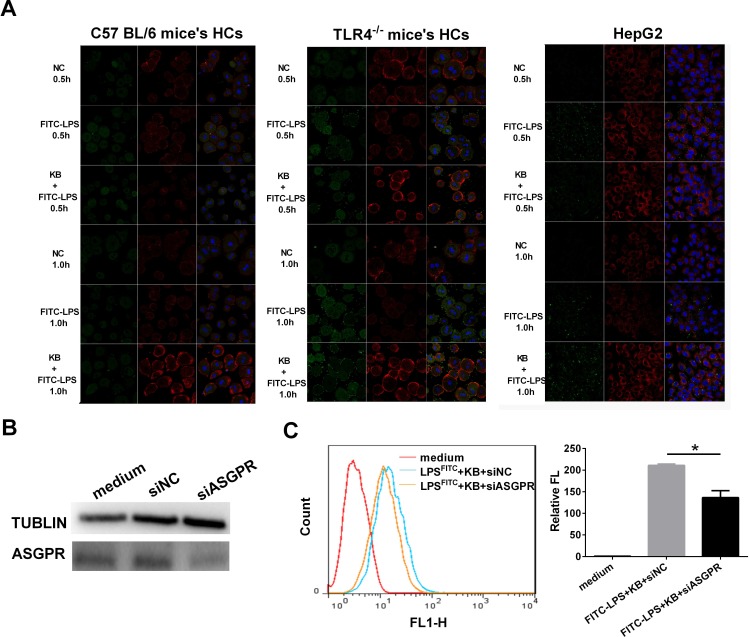
ASGPR knockdown dampens KB-induced LPS uptake in murine hepatocytes **A.** HCs from WT or TLR4^−/−^ mice and HepG2 were untreated (NC) or treated with FITC-LPS (1 μg/ml) alone or preincubated with KB (15 μM). FITC-LPS (green) and ASGPR (red) were detected in HCs at 0.5 and 1 h. **B.** HepG2 cells were transfected with negative control siRNA (siNC) or ASGPR siRNA (siASGPR). ASGPR siRNA interference efficiency was evaluated by western blotting. **C.** HepG2 cells were transfected with siNC or siASGPR as in **B.** and further treated with FITC-LPS (1 μg/ml) preincubated with KB (15 μM) for 1 h. LPS uptake was measured by flow cytometry and expressed as relative fluorescence (Relative FL). *P < 0.05

As demonstrated by immunoblotting, increased FITC-LPS uptake was accompanied by elevated ASGPR expression in HCs from WT mice, TLR4^−/−^ mice and HepG2 cells (Figure 5A). In addition, we transfected ASGPR siRNA into HepG2 cells and observed dampened KB-induced LPS uptake (Figure 5B-5C). These data indicated that ASGPR was selectively targeted by KB to mediate LPS uptake in hepatocytes.

### KB directly binds ASGPR and mediates LPS internalization

**Figure 6:**
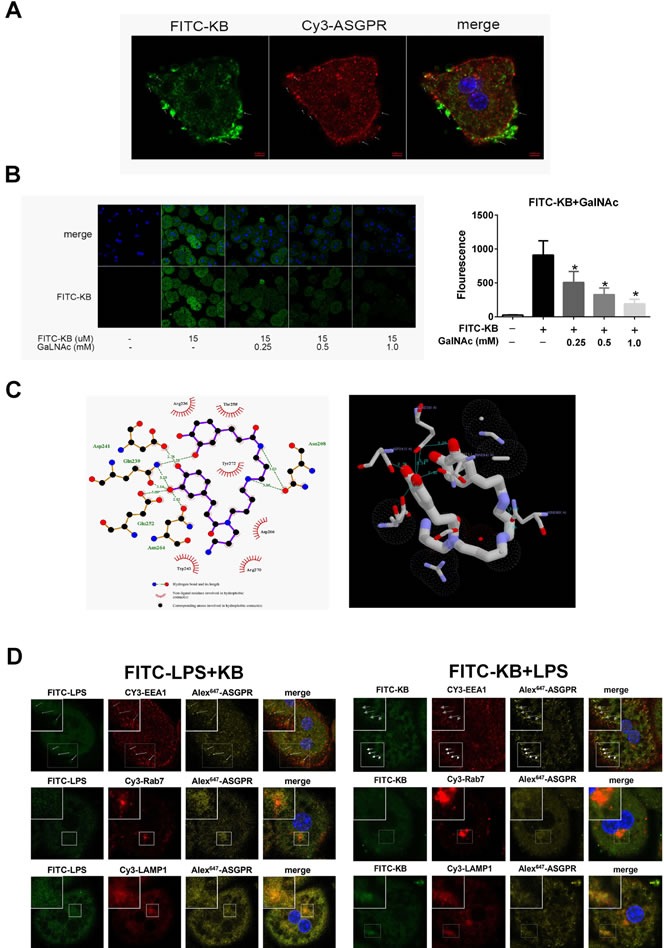
KB colocalizes with ASGPR and competes with GalNAc in hepatocytes **A.** HCs from WT mice were treated with FITC-KB (15 μM) for 30 min. KB (green) and ASGPR (red) colocalization was detected by immunofluorescence. **B.** C57 BL/6 HCs were incubated with treated with KB (15 μM) for 1 h with or without GalNAc (0.25, 0.5 or 1.0 mM). FITC-KB was detected by confocal microscopy and MFI was quantified from mean fluorescence (*n* > 30). *P < 0.05 *vs*. FITC-KB. **C.** Molecular docking was performed with AutoDockVina. The detailed binding domain (left) and three-dimensional binding graph (right) are presented. **D.** HCs from TLR4−/− mice were treated with 1 μg/ml FITC-LPS preincubated with 15 μM KB (left) or with 1μg/ml LPS preincubated with 15 μM FITC-KB (right) for 1 h. Colocalization of FITC-LPS (green), FITC-KB (green) and ASGPR (yellow) with EEA1 (red), Rab7 (red) and LAMP1 (red) are presented as fluorescence images captured by a laser scanning confocal microscope.

To determine whether KB directly interacted with ASGPR, we assessed FITC-KB and ASGPR colocalization. KB colocalized with ASGPR in hepatocytes (Figure 6A). Previous studies showed that GalNAc is a natural ASGPR agonist in HCs [31, 32]. Therefore, we used GalNAc to compete with free FITC-KB for interaction with ASGPR. FITC-KB uptake by HCs in WT mice was reduced with increasing GalNAc dosage, suggesting that KB competed with GalNAc for ASGPR binding in murine hepatocytes (Figure 6B). Molecular docking analysis [30] showed that KB binds the carbohydrate recognition domain of ASGPR with a predicted binding energy of −5.2 kcal/mol. Hydrogen bonding and hydrophobic interaction was predicted between KB and ASGPR (Figure 6C). These binding sites and bonds closely resembled the predicted interaction between GalNAc and ASGPR. We also observed that ASGPR distributed with the KB-LPS complex after uptake. ASGPR colocalized with the complex in early endosomes, late endosomes and lysosomes, indicating a possible endosome-dependent route of intracellular degradation (Figure 6D).the conjugation of KB with LPS might be a priming condition before uptake by ASGPR.

### KB inhibits LPS-triggered inflammation and protects hepatocytes while promoting LPS uptake

**Figure 7:**
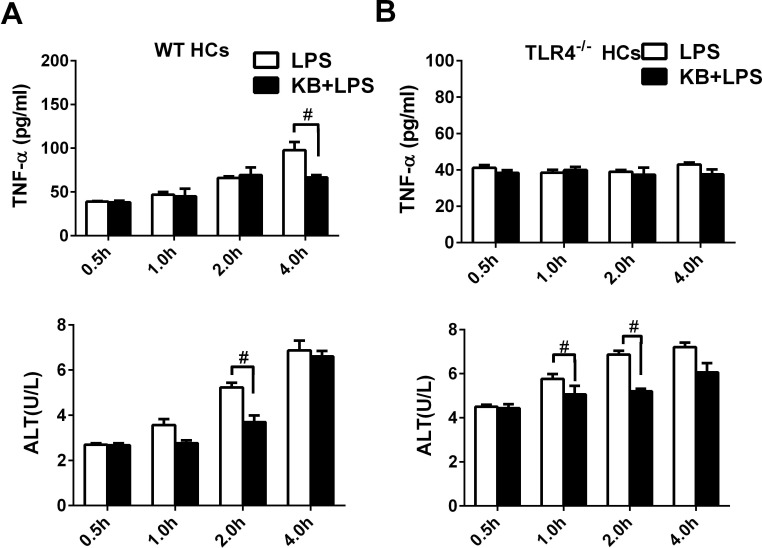
KB reduces inflammation caused by LPS and protects HCs HCs from WT **A.** or TLR4^−/−^ **B.** mice were treated with LPS (1 μg/ml) preincubated with or without KB (15 μM). Supernatants were collected at 0.5, 1, 2 or 4 h. TNF-α levels were detected by ELISA and ALT activity was detected *via* automatic biochemical analyzer. Data are the result of three replicates and are expressed as means ± SD. #*P* < 0.01 *vs.* LPS group.

To assess the impacts of increased LPS uptake on cell viability, supernatant ALT and TNF-α levels were detected in cultured primary HCs upon LPS stimulation. ALT activity and TNF-α levels, as measured from 0.5 to 4 h, increase following LPS stimulation. However, KB effectively inhibited the release of ALT and TNF-α induced by LPS (Figure 7A). Similar inhibitory effects were observed in HepG2 cells (Figure S4). In hepatocytes isolated from TLR4^−/−^ mice, LPS was unable to induced TNF-α production, probably due to the lack of TLR4 dependent signaling. However, LPS treatment still elevated ALT activity in TLR4^−/−^ hepatocytes, and KB reduced ALT production, demonstrating potential hepatocyte protection by KB (Figure 7B).

### KB activity differs from that of GalNAc in neutralizing LPS

**Figure 8:**
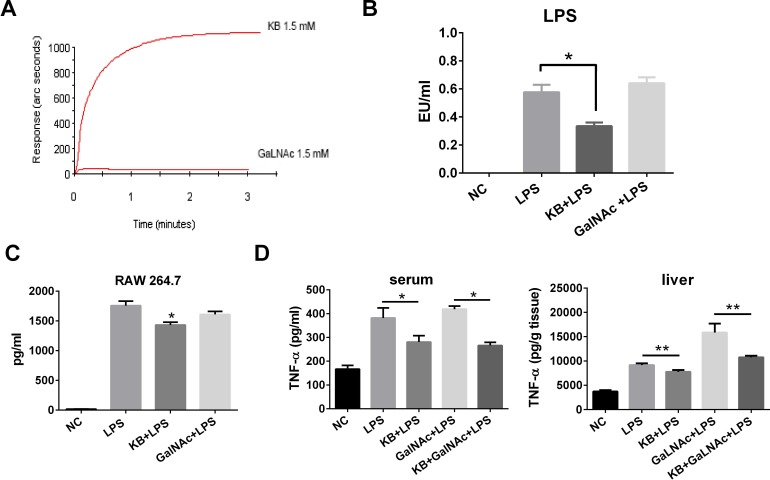
KB directly binds and inhibits LPS while GalNAc does not **A.** Binding of KB (1.5 mM) and GalNAc (1.5 mM) with lipid A, the active center of LPS, was examined by bio-sensor. **B.** LPS (1 ng/ml) neutralization by GalNAc (4.5 μM) or KB (4.5 μM) was examined by limulus test. *P < 0.05. **C.** RAW 264.7 cells were incubated with LPS (100 ng/ml) alone or with KB (100 μM) or GalNAc (100 μM) for 4 h. TNF-α levels in the supernatant were examined by ELISA. *P < 0.05 *vs* LPS. **D.** C57 BL/6 mice were injected with sterile NS or LPS (1 mg/kg) preincubated with NS, KB (3.6 mg/kg), GalNAc (100 mg/kg) or KB+ GalNAc. TNF-α levels in blood and liver tissues were examined at 4 h. *P < 0.05, **P < 0.01.

KB and GalNAc both bind ASGPR. However, their effects on LPS might be different, as KB was protective in LPS-treated hepatocytes while GalNAc is an LPS sensitizer. We used affinity detection to assess direct binding between KB or GalNAc and LPS. KB bound *E. coli* LPS with high affinity, while almost no measurable binding occurred between GalNAc and LPS (Figure 8A). In addition, KB directly neutralized LPS *in vitro* in the LAL test, while GalNAc did not (Figure 8B), and KB effectively suppressed TNF-α release in LPS-stimulated RAW 264.7 cells. GalNAc failed to reduce TNF-α production, consistent with the affinity and LAL tests (Figure 8C). In LPS injected mice, KB reduced circulating TNF-α levels and inhibited TNF-α production in liver homogenates, although it induced LPS uptake by hepatocytes. In contrast, GalNAc treatment did not affect circulatory LPS, but did increase TNF-α production in liver homogenates (Figure 8D).

## DISCUSSION

The host response to bacterial LPS often drives intrinsic defense mechanisms against bacterial invasion [31, 32]. Although free LPS is metabolized in the liver *via* TLR4-dependent mechanisms, the pathways that mediate hepatic uptake of conjugated LPS remain unclear. We found that a novel small molecular LPS antagonist, KB, not only mediated the biological effects of LPS through direct binding and neutralization, but also accelerated its blood clearance by enhancing TLR4-independent LPS uptake in hepatocytes. KB may upregulate ASGPR, a membrane transporter in hepatocytes that mediates internalization and metabolism of conjugated LPS.

KB is a novel LPS antagonist that reliably treats sepsis [28, 29, 33]. We found that KB effectively reduced circulating LPS and TNF-α levels in LPS-challenged mice through direct blockade of both LPS and LPS-induced inflammation. Similar results were reported for other LPS neutralizers, such as HDL, PMB, BPI and LL-37 [24, 34, 35]. Recent studies have investigated the consequences of LPS neutralization on LPS distribution and clearance. For example, the human cationic antimicrobial protein, LL-37, reportedly enhanced LPS uptake by liver sinusoidal endothelial cells without inducing pro-inflammatory reactions [24]. In contrast, another intrinsic LPS carrier, HDL, interfered with hepatic LPS uptake [10, 36, 37]. In our study, we observed similar activity of KB with LPS-neutralizing LL-37, which accelerated serum clearance of LPS while selectively increasing its accumulation in liver homogenates.

The liver is the primary organ responsible for LPS adsorption and removal. Within the liver, HCs and KCs appear to be most critically involved in mediating hepatic LPS uptake [38, 39]. LPS can be found in HCs within five min following intravenous injection and approximately 75% of injected LPS is directly removed by HCs [40–42]. KCs are a subtype of macrophages that reside in the liver, and LPS absorbed by KCs is further degraded by AOAH-mediated deacylation. However, excessive LPS can induce KCs to release cytokines and chemokines and cause liver injury [43, 44]. In our study, KB selectively increased LPS uptake in HCs while inhibited KCs from internalizing LPS. KB still induced hepatic LPS uptake when KCs were depleted. In contrast, others have shown that HDL-conjugated LPS was still distributed in KCs despite lower numbers of these cells [10]. Such results may be explained by predominant SR-B1 (HDL receptor) expression in KCs which internalize HDL-conjugated LPS [37]. Importantly, while KB selectively induces hepatocyte uptake of LPS, it also protects liver tissue by inhibiting KC-induced inflammation.

Generally, the CD14/TLR4/MD2 recognition complex is required for free LPS uptake in HCs and KCs [45, 46]. However, for conjugated LPS, the LPS-neutralizing peptide, LL-37, might mediate LPS uptake in human LSECs *via* interaction with cell-surface heparan sulfate proteoglycans and TLR4 is not required for LL-37-LPS complex uptake [24]. In our study, KB directly bound LPS, interfering with routine TLR4-dependent LPS uptake in KCs and RAW 264.7 cells. However, KB promoted LPS uptake in HCs isolated from both WT and TLR4^−/−^ mice, suggesting that KB-conjugated LPS was absorbed independently of TLR4. Therefore, we assessed whether KB affected other LPS uptake-related receptors in HCs [13, 47]. We found that KB did not affect the expression of major scavenger receptors (SR-A, SR-B1 and MARCO) that commonly mediate the binding and internalization of LPS in HCs and in KCs. In addition, KB did not alter mRNA levels of transferrin receptor or LOX-1, a hepatocyte cell-surface receptor for oxidized low-density lipoprotein (ox-LDL). However, ASGPR was selectively upregulated in both live tissues and isolated hepatocytes treated with KB alone or with the KB-LPS complex. We also discovered that KB-mediated LPS internalization in hepatocytes was accompanied by ASGPR upregulation. Moreover, ASGPR knockdown impaired LPS uptake in hepatocytes. These results together suggest that KB may upregulate ASGPR as an alternative uptake receptor for conjugated LPS.

ASGPR, also known as the Ashwell receptor, is a highly potent C-type lectin receptor expressed abundantly on hepatocytes. ASGPR internalizes serum glycoproteins with the galactose (Gal) or N-acetylgalactosamine (GalNAc) residue through receptor-mediated endocytosis [48, 49]. Although direct involvement of ASGPR in hepatic uptake of LPS was not reported previously, recent studies showed that ASGPR interacts with extracellular pathogens to induce their phagocytosis by HCs [50, 51]. In our study, KB was internalized in hepatocytes with or without LPS, which indicated that LPS uptake might be mediated by direct KB recognition. GalNAc is a high-affinity ASGPR ligand utilized for the delivery of proteins, peptides and nucleic acids to hepatocytes through ASGPR binding. We observed that GalNAc dose-dependently suppressed KB uptake, indicating that GalNAc may competitively inhibit KB. This was supported by our molecular docking study, as KB was predicted to directly bind ASGPR, and the predicted binding sites between KB and ASGPR were identical with those of GalNAc [52]. ASGPR can mediate the internalization of organic molecules containing galactosyls residues, and participates in cellular apoptosis as well as endocytosis and removal of LDL and chylomicron [50]. ASGPR mediates glycoprotein internalization and degradation at lysosomes. We found that ASGPR colocalized with the KB-LPS complex within early endosomes, late endosomes and lysosomes, indicating that ASGPR might also mediate the intracellular metabolism of conjugated LPS in hepatocytes *via* the endosome-lysosome system.

TLR4 is commonly regarded as the key LPS receptor in triggering the host pro-inflammatory response. However, a previous study reported that TLR4-knockout HCs still absorb LPS and exhibit increased cellular damage. In our study, TLR4-knockout HCs had dampened pro-inflammatory responses upon LPS treatment. However, this did not suppress cellular damage, indicating that free LPS may be still internalized. We found that the combination of KB and LPS promotes LPS absorption by HCs, but inhibits absorption by KCs. KB also inhibits TNF-α release by HCs of WT mice. In HCs of TLR4-knockout mice, which did not release TNF-α due to TLR4 deficiency, KB still protected liver tissue by inhibiting ALT release. These data suggest that KB binds LPS and attenuates its cytotoxicity. Therefore, enhanced LPS uptake does not cause cellular injury. While KB and GalNAc both bound ASGPR, their effects on LPS-induced inflammation in HCs appeared to be different. In our study, GalNAc did not interact with or inhibit LPS even in high concentrations, and thus did not protect HCs from injury induced by LPS-triggered inflammation. KB not only bound ASGPR, but also conjugated LPS to protect against cell damage. In addition, we found that KB pretreatment did not enhanced LPS uptake in HCs. Such results also suggest that the conjugation of KB with LPS should be a priming condition to mediate the uptake. Nevertheless, it is possible that other mechanisms may be involved which worth further study in the future.

In summary, KB forms a complex with LPS, inhibits LPS absorption by KCs, and may promote LPS absorption in HCs by activating and upregulating ASGPR expression. In HCs, KB accelerates LPS internalization while inhibiting inflammation, thus protecting liver tissue. Future work is needed to further determine the cellular destination of the internalized KB-LPS complex as well as its specific degradation mechanism in HCs, and may provide additional support for the application of KB as a promising candidate drug in sepsis treatment.

## MATERIALS AND METHODS

### Animals

Wide type male C57BL/6 mice (9-12 weeks) were purchased from HFK Bioscience (Beijing, China). TLR4^−/−^ C57BL/10 mice (9-12 weeks) were purchased from the Model Animal Research Center of Nanjing University (Nanjing, China). Mice were kept under standard specific pathogen free conditions with free access to food and water. All animal experiments were performed in accordance with the National and Institutional Guidelines for Animal Care and Use and approved by the institutional Animal Ethic Committee of the Third Military Medical University.

### Chemicals and reagents

*E. coli* LPS (serotype O55:B5), L-Glutamine, BSA and HEPES were purchased from Sigma-Aldrich (MA, USA). Alexa Fluor^®^ 488- and 568-conjugated LPS (*E. coli* O55:B5) were purchased from Life Technologies (OR, USA). KB- and FITC-conjugated KB were synthesized at Sichuan University. GalNAc was purchased from Aladdin Corporation (Shanghai, China). OptiPrep™ Density Gradient Medium was purchased from Axis-Shield (Oslo, Norway). MACS, anti-F4/80-PE and anti-PE microbeads were purchased from Miltenyi Biotec (Auburn, CA). SYBR^®^Green realtime PCR Master Mix-plus- was from TOYOBO (Osaka, Japan). Human and mouse TNF-α ELISAs were purchased from eBioscience (San Diego, CA). X-treme GENE siRNA Transfection Reagent was from Roche (Mannheim, Germany). Antibodies against CD31 and glial fibrillary acidic protein (GFAP) were obtained from Biolegend (San Diego, CA). Antibodies against F4/80, cytokeration 18, TLR4 and LAMP1 were from Abcam (Cambridge, UK). Cy3, Alexa Fluor 647 or biotin-labeled secondary antibody against IgG (H+L) was from Beyotime (Jiangsu, China). Antibodies against asialoglycoprotein receptor (ASGPR), EEA1, Rab7 and ASGPR1 siRNA were from Santa Cruz Biotechnology (CA, USA). All chemicals used were analytical grade or best commercially available.

### Affinity biosensor assay

Direct binding of GalNAc and KB to LPS was detected *via* affinity sensor and as described previously [29]. Briefly, 1 μl GalNAc or KB (1.5 mM) was added to the LPS cuvette containing 49 μl 0.02 M PBS. The binding reaction was incubated for 3 min and immobilized on the reacting surfaces of cuvettes in an IAsys plus affinity biosensor (Farfield, Cheshire, UK). Unbound GalNAc or KB was then dissociated with PBS and regenerated with 0.01 M HCl. The whole process of reaction, including binding, dissociation and regeneration, was recorded by the biosensor, and calculated using the FASTplot software package (Farfield, UK).

### Limulus amebocyte lysate (LAL) assay

For *in vitro* LPS neutralization detection, GalNAc or KB (4.5μM) were incubated with an equal volume of LPS (1ng/ml) at 37°C for 30 min, and then detected using an ATI 320-06 kinetic tube reader (Lab Kinetics Ltd, Bruton, UK). For *in vivo* detection, blood samples were obtained *via* heart punctures and centrifuged to isolate plasma. Plasma LPS was detected using the LAL assay.

### Fluorescence detection of LPS organ distribution in injected mice

FITC-LPS (1mg/kg) was incubated with KB (3.6 mg/kg) or NS at 37°C for 30 min and injected intravenously. Mice were sacrificed 1, 4, 8 or 24 h after LPS injection. FITC-LPS fluorescence intensity in serum and tissue homogenates was detected at 488 nm *via* a microplate reader (Thermo).

### Immunofluorescence detection of hepatic cell LPS uptake in frozen tissue sections

FITC-LPS (1mg/kg) was incubated with KB (3.6mg/kg) or NS at 37°C for 30 min and injected intravenously into mice. 1 h later., the mice were sacrificed by anesthesia, and frozen sections of liver tissue were prepared immediately, fixed with 4% paraformaldehyde for 15min, sealed with 3% BSA at 4°C for 30min in dark, and stained with antibodies for cytokeratin 18, F4/80, GFAP and CD31 at 4°C overnight, followed by staining with Cy3 labeled antibody and DAPI (nuclear staining). Fluorescence images were captured *via* a ZEISS 780 laser confocal microscope (Germany).

### Isolation of primary hepatocytes and Kupffer cells

Primary hepatocytes were isolated by an improved two-step collagenase infusion [53, 54]. Generally, mice fasted for 16 h were sacrificed and injected with buffer solution through the retro-hepatic postcaval vein after ligation of the precaval vein, followed by collagenase IV injection. Then the whole liver was removed 6 min later, cut into pieces and immersed in perfusion buffer (Hank's Balanced Salt Solution with 2 mM calcium chloride, 1 mg/ml glucose, 0.2 mg/ml collagenase IV and 10 μg/ml DNase I) and incubated at 37°C for 15 min. Cellular sediments were obtained by filtration and centrifuged. Cell pellets were washed twice in 5ml PBS with 1% BSA, followed by gradient centrifugation with 10% Optiprep (50 g, 4°C, 10 min). HCs were re-suspended in DMEM supplemented with 10% FBS, 2 mM L-glutamine, 0.01 μg/ml EGF primary liquid, 15 mM HEPES, 100 U/ml penicillin and 100 mg/ml streptomycin, and cultured in 6-well plates or confocal dishes pre-coated with rat tail collagen in a humidified incubator with 5% CO_2_ at 37°C.

An improved method was used for Kupffer cell isolation. Total hepatic cell preparations were performed as described above. Supernatant was obtained and gradient centrifugation was employed (400 g, 4°C, 10min) to get cellular sediments. Kupffer cells were purified *via* immune-magnetic bead separations (IMB). Isolated Kupffer cells were inoculated in 6-well plates and cultured in DMEM with 10% FBS, 100 U/ml penicillin and 100mg/ml streptomycin for 2 h in a humidified incubator with 5% CO_2_ at 37°C.

### Cell culture and treatment

HepG2 cells, RAW 264.7 cells (ATCC, Manassas, VA, USA), murine primary hepatocytes and Kupffer cells were cultured in high glucose DMEM supplemented with 10% FBS at 37°C in a 5% CO_2_ humidified incubator. Cell viability was measured by Trypan blue staining. Cell density was measured using a TC20 cell counter (Bio-rad).

### Immunofluorescence detection of hepatocyte LPS uptake

FITC-KB or FITC-LPS was incubated with LPS or KB for 30 min and added to TLR4^−/−^ HCs (2×10^5^/ml) for 1 h. Cells were fixed in 4% paraformaldehyde, permeabilized by 0.01% Triton and incubated with primary antibodies for ASGPR1, EEA1, Rab7 and LAMP1, followed by staining with Alexa Fluor 647 or CY3-labled anti IgG (H+L). Nuclei were stained with DAPI and fluorescence images were captured with a ZEISS 780 laser confocal microscope (Germany).

### Flow cytometry

Cells were treated as indicated, stained with FITC-LPS for 1 h and detected using a FACSCalibur (BD Biosciences, USA) flow cytometry system with the test condition of He-Ne-laser (λEx = 488nm). 10,000 cells were tested for each independent sample.

### Quantitative RT-PCR

HepG2 cells and mouse primary hepatocytes were cultured in 6-well plates and treated as indicated. Cell pellets were washed and total RNA was extracted with the RNA simple total RNA kit (TIANGEN Biotech, China). The ReverTra Ace qPCR RT kit (TIANGEN Biotech, China) was used to reversely transcribe total RNA into cDNA according to the manufacturer's instructions. cDNA templates were mixed with SYBR Green PCR mastermix (TOYOBO) and primers for mouse TLR4, ASGPR, MARCO, SR-B1, Tfrc, SR-A, LOX-1 and β-actin (Table S1). Quantitative real-time PCR was performed with an iCycler Thermal Cycler (Bio-Rad).

### Western blot

Mouse hepatocytes or HepG2 cells (2×10^5^ cells/ml) cultured on 6-well plates were washed in PBS three times and cell pellets were collected. For hepatic tissues, dissected liver tissue was homogenized. Cell pellets or tissue homogenates were lysed with RIPA solution containing phosphatase and proteinase inhibitors. Total plasma protein was collected and quantified by BCA. Plasma proteins were separated by SDS-PAGE and transferred onto PVDF membranes (Millipore, USA). Blots were blocked with 5% BSA for 1 h and incubated with primary antibodies (1:1000) for ASGPR, followed by HRP-labeled goat anti-rabbit IgG (H+L) (1:2000) for 1 h at room temperature. The ChemiDocTM XRS + system (Bio-Rad, USA) was used to detect blots, and results were analyzed using Image Lab software (Bio-Rad, USA).

### siRNA transfection

X-treme GENE siRNA Transfection Reagent was used to transfect HepG2 cells according to the manufacturer's protocol. HepG2 cells (2×10^5^ cells/ml) were cultured overnight and transfected with negative control siRNA or ASGPR siRNA mixed in X-treme GENE siRNA Transfection Reagent diluted in Opti-MEM^®^ I Reduced Serum Media. The mixture was added to cultures for 6 h after being gently blended, and was replaced with complete culture medium at 37°C, 5% CO_2_ for 36 h before further treatment.

### Molecular docking

Molecular docking was performed with AutoDockVina [30]. The structure of the asialoglycoprotein receptor H1 subunit was retrieved from the Protein Data Bank (PDB ID: 1DV8). All rotatable kukoamine B bonds were made rotatable. A grid box with a size of 20 × 18 × 20 angstrom and a center of 6.46 × −4.47 × 10.41 points, which included the carbohydrate recognition domain, was created with AutoDockTools GUI for a conformational search [55]. All other parameters were set to their default settings. The docking results were analyzed using AutoDockTools and LigPlot+ [56].

### ELISA detection of ALT and TNF-α

HCs isolated from WT or TLR4^−/−^ mice and HepG2 cells were cultured and treated as indicated. Supernatants were collected at 0.5, 1, 2 and 4 h after LPS treatment. The Beckman coulter AU480 automatic biochemical analyzer (Beckman Coulter, Inc., USA) was used to detect ALT. TNF-α was detected by ELISA with the human or mouse TNF ELISA Ready-Set-Go kit (eBioscience, CA) according to the manufacturer's instructions.

### Statistical analysis

Data were represented as means ± SD and examined by one-way ANOVA or Student's *t*-test. All statistical tests were two-sided and *P* < 0.05 was considered statistically significant.

## SUPPLEMENTARY MATERIAL FIGURES


