# Supplementary material for: Kukoamine B promotes TLR4-independent lipopolysaccharide uptake in murine hepatocytes
Source: Oncotarget. 2016 Aug 15;7(36):57498–513. doi: 10.18632/oncotarget.11292 (PMC5295368; doi:10.18632/oncotarget.11292)
Supplement: Supplementary file 1 [file oncotarget-07-57498-s001.pdf]

Kukoamine B promotes TLR4-independent lipopolysaccharide uptake in murine hepatocytes

Supplementary Material

Table S1: Quantitative real-time PCR primers.

mouse ASGPR	Sense: 5'- CCAGCTCTGAGTCTGTTAGCC-3'
	Antisense: 5'- CAGATCTTCCCGGAGTTGGG-3'
mouse MARCO	Sense: 5'- AGAAAGGGAGACACTGGAAGC -3'
	Antisense: 5' -CCTCTGGAGTAACCGAGCAT-3'
mouse SR-B1	Sense: 5'- ATCTGGTGGACAAATGGAA -3'
	Antisense: 5'- GAAGCGATACGTGGGAAT -3';
mouse β-actin	Sense: 5'-CTGTCCCTGTATGCCTCTG-3'
	Antisense: 5'- ATGTCACGCACGATTTCC-3'.
mouse TLR4	Sense: 5'-TTTATTCAGAGCCGTTGG-3'
	Antisense: 5'- AGTTGCCGTTTCTTGTTC-3'
mouse TFRC	Sense: 5'- TCATGAGGGAAATCAATGATCGTA-3'
	Antisense: 5'- GCCCCAGAAGATATGTCGGAA-3'
mouse SR-A	Sense: 5'- ATTGGGAAATGAAGAACTGC-3'
	Antisense: 5'- GGACTGACGAAATCAAGGAA-3
mouse LOX-1	Sense: 5'- GCATGAATTTGGAAATGACTTTTGA-3
	Antisense: 5'- TCTGCCCTTCCAGGATACGA-3'

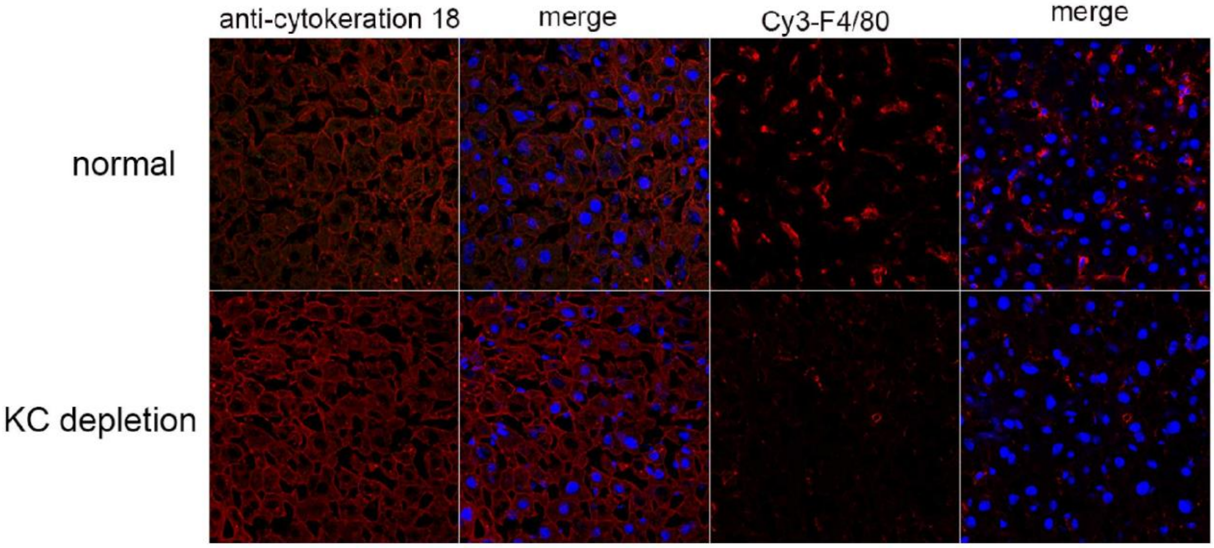

Figure S1: KCs in liver tissue were depleted by Clophosome. C57 BL/6 mice were injected with Clodronate liposomes (140 μl/20 g) or sterilizing NS for 48 h. Frozen liver sections were prepared and markers for HCs (cytokeratin 18) and KCs (F4/80) were detected by confocal microscopy (objective×40). Nuclei were stained by DAPI (blue).

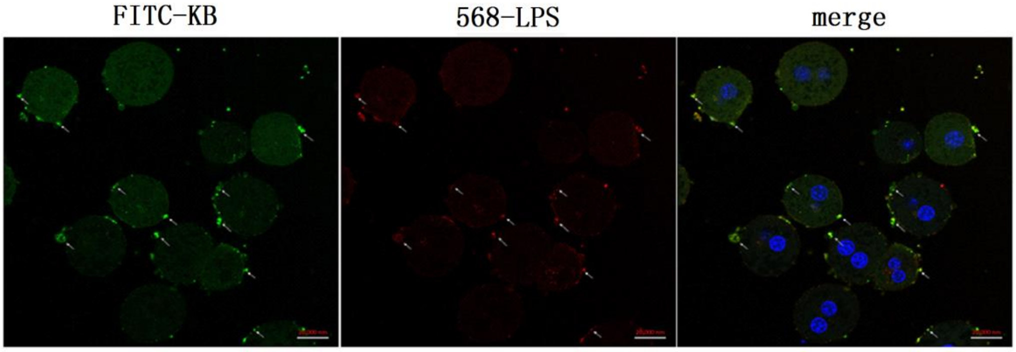

Figure S2: Colocalization of KB with LPS in cultured HCs. HCs from WT mice were treated with Alexa Fluor®568-conjugated LPS (1 μg/ml) preincubated with FITC-KB (15 μM) for 30 min *in vitro*. FITC-KB (green) and Alexa Fluor®568-conjugated LPS (orange-red) colocalization was detected on HC cell membranes by confocal microscopy 30 min later.

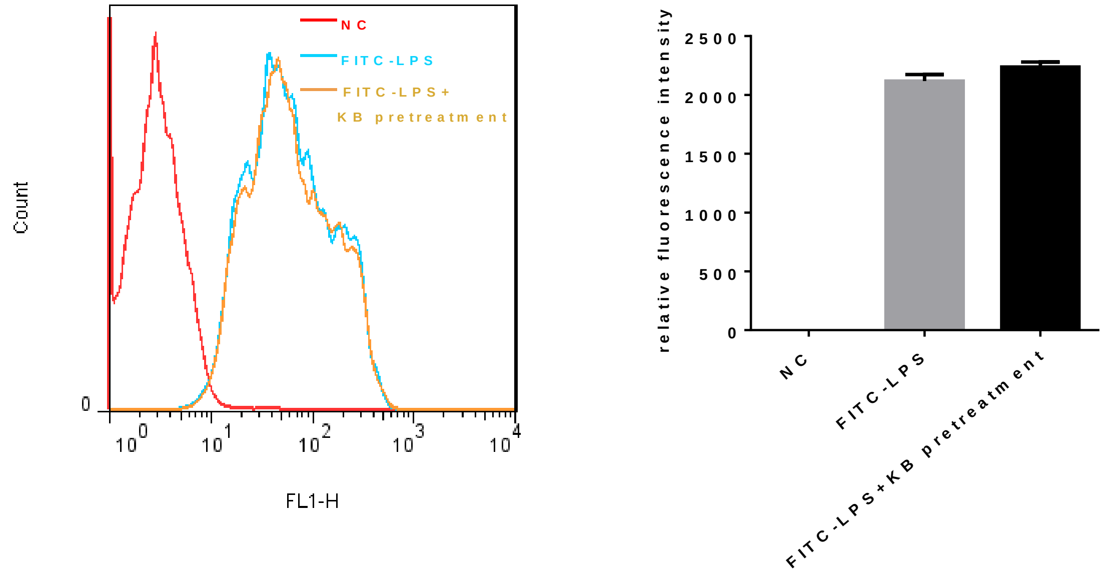

Figure S3: Pretreatment with KB did not affect the uptake of free LPS in HCs. HCs from WT mice were untreated (NC), or treated with FITC-LPS (1 µg/ml) or treated with FITC-LPS after preincubation by KB (15 µM) for 2 h. The uptake was detected by flow cytometry 1h after LPS treatment.

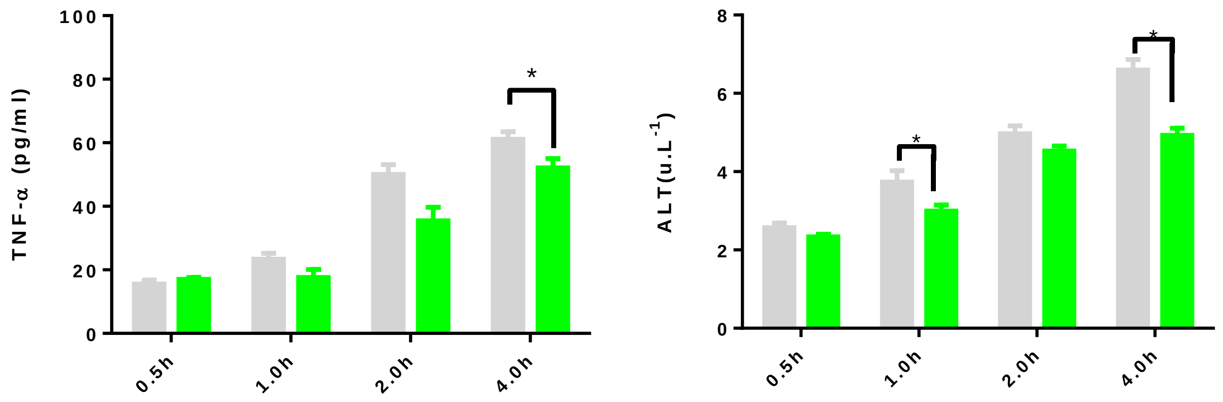

Figure S4: KB inhibits the release of TNF-α and ALT from LPS-stimulated HepG2 cells. HepG2 cells were treated with LPS (1 μg/ml) preincubated with or without KB (15 μM). Supernatants were collected at 0.5, 1, 2 or 4 h. TNF-α levels were detected by ELISA, and ALT activity was detected via automatic biochemical analyzer. *P<0.01.
